# A novel multiwalled carbon nanotube–cyclodextrin nanocomposite for solid-phase microextraction–gas chromatography–mass spectrometry determination of polycyclic aromatic hydrocarbons in snow samples

**DOI:** 10.1007/s00604-023-05799-8

**Published:** 2023-05-12

**Authors:** N. Riboni, F. Bianchi, M. Scaccaglia, F. Bisceglie, A. Secchi, C. Massera, P. Luches, M. Careri

**Affiliations:** 1 University of Parma, Department of Chemistry, Life Sciences and Environmental Sustainability, Parco Area delle Scienze 17/A, 43124 Parma, Italy; 2 University of Parma, Center for Energy and Environment (CIDEA), Parco Area delle Scienze 42, 43124 Parma, Italy; 3 Nanoscience Institute, CNR, via G. Campi 213/A, 41125 Modena, Italy

**Keywords:** Cyclodextrins, Carbon nanotubes, Solid-phase microextraction, Polycyclic aromatic hydrocarbons, Gas chromatography–mass spectrometry

## Abstract

Novel solid-phase microextraction coatings based on the use of multiwalled carbon nanotube–cyclodextrin (MWCNT-CD) nanocomposites were developed for the determination of 16-priority polycyclic aromatic hydrocarbons at ultratrace levels in snow samples. The performance of both β- and γ-CD was tested to increase the detection capabilities towards the heaviest and most lipophilic compounds, i.e., five- and six-ring PAHs. To facilitate the interactions of MWCNTs with CDs, an oxidation procedure using both HNO_3_ and H_2_O_2_ was applied, obtaining superior results using MWCNTs-H_2_O_2_-γ-CD fiber. Detection and quantitation limits below 0.7 and 2.3 ng/L, RSD lower than 21%, and recoveries of 88(± 2)–119.8(± 0.4)% proved the reliability of the developed method for the determination of PAHs at ultratrace levels. The complexation capability of the γ-CD was also demonstrated in solution by NMR and fluorescence spectroscopy studies and at solid state by XRD analysis. Finally, snow samples collected in the ski area of Dolomiti di Brenta were analyzed, showing a different distribution of the 16 priority PAHs, being naphthalene, phenanthrene, fluoranthene, and pyrene the only compounds detected in all the analyzed samples.

**Graphical Abstract:**

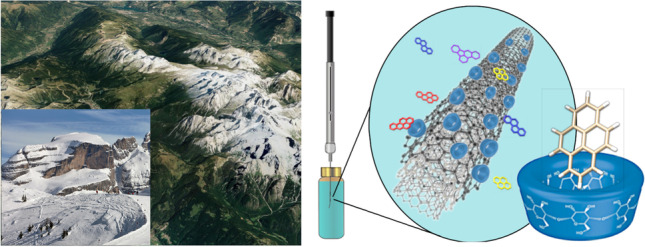

**Supplementary information:**

The online version contains supplementary material available at 10.1007/s00604-023-05799-8.

## Introduction

The determination of trace level pollutants in complex matrices such as environmental samples, biofluids, and tissues is considered one of the most challenging tasks in analytical chemistry. Therefore, sample pretreatment is a crucial step to ensure accurate and reliable results, targeting selected analytes while minimizing the presence of interfering compounds [1].

Extraction techniques based on the use of a solid sorbent have gained increasing interest, thus featuring a wide range of sorbents for improved, selective, and effective analyte recovery. Among the different commercially available sorbents, silica, zeolites, activated carbon, polymeric resins, polymer-coated porous materials, and molecular sieves are the most applied. However, they present several drawbacks, encompassing low enrichment capabilities, reduced selectivity, limited resistance to temperature, pH, and ionic strength of the media [1, 2]. To overcome these limitations, new compound-specific and class-specific materials have been proposed as sorbents for solid-phase extraction (SPE) [2, 3], dispersive solid-phase extraction (DSPE) [4], and solid-phase microextraction (SPME) [5–7]. The presence of functional groups on the surface of the sorbent materials is a key parameter to promote selective enrichment of analytes [2, 6].

Thanks to enhanced surface area/volume ratios, engineered morphologies, and the possibility of selective extraction, nanomaterials and nanostructured sorbents have opened new challenges in sample pretreatment [3]. The use of these materials, including carbon-based sorbents, metal organic frameworks, magnetic nanoparticles, and polymer nanosorbents [3, 4, 6], results in increased adsorption capacity, improved preconcentration factors, and reduced extraction time.

Because of their hydrophobic nature and the possibility to establish π-π, CH-π, and hydrophobic interactions, CNTs have been proposed as nanosorbent for the extraction/removal of a wide range of hydrophobic compounds from complex matrices [5, 6, 8–10]. To increase selectivity towards more polar analytes, chemical and physical functionalization can be carried out by introducing moieties such as carboxylic groups, hydroxyls, amines, or amides onto the outer walls of the CNTs [3, 11]. Improved extraction performance can be obtained by the insertion of ionic liquids, polymeric nanoparticles, and cyclodextrins (CDs) [3, 6, 11].

Supramolecular receptors provide unique features in terms of selectivity, being the inclusion of the analytes based both on the formation of specific interactions during complexation and on the host-guest shape complementarity based on the lock-key principle. Several receptors have been proposed for sample pretreatment including CDs [2, 12, 13], calixarenes [2, 12], and cavitands [14–16].

CDs can form host-guest complexes with different compounds, such as dyes, ions, and volatile and semivolatile organic compounds: in particular, both β- and γ-CDs demonstrated excellent inclusion performances towards polycyclic aromatic hydrocarbons (PAHs) [17–21]. Other advantageous features rely on the low price compared to other designed receptors, negligible environmental impact, and near-zero toxicity.

In a recent study [9], we developed a helical MWCNT-based SPME coating for the gas chromatography–mass spectrometry (GC-MS) determination of PAHs at ultratrace levels in Antarctic snow samples. Reduced sample handling and a significant reduction in sample volume address the analytical challenges of high temporal resolution records in paleoclimatic investigations. In this context, screening the presence of environmental contaminants like PAHs at very low concentration levels is of paramount importance to verify both the anthropogenic impact and transport mechanisms. The material previously developed was characterized by excellent performance in terms of enrichment capabilities and sensitivity; however, the detection of high-molecular weight compounds proved to be very challenging, being their concentrations lower than those of the lighter and more hydrophilic PAHs.

In this study, novel nanocomposite materials based on β- and γ-CD-functionalized COOH-MWCNTs are proposed to increase the detection capabilities towards five- and six-ring PAHs in snow samples, thus allowing a deeper investigation on the synergistic role of MWCNTs and CDs in the extraction/complexation of the investigated analytes. To our knowledge, this study reports for the first time the application of the multiwalled cyclodextrin nanocomposite materials for the extraction of PAHs from snow samples.

## Experimental section

### COOH-MWCNT functionalization and characterization

Chemical oxidation of COOH-MWCNTs was performed using either HNO_3_ or H_2_O_2_ based on published procedures with proper modifications [22, 23] as reported in ESM.

### X-ray photoemission spectroscopy (XPS)

To understand the evolution of surface chemistry with the different functionalization treatments, XPS measurements were performed in an ultrahigh vacuum chamber using Al Kα photons. C 1 s and O 1 s spectra were fitted using six Voigt-shaped functions and a Shirley-type background (see ESM).

### Fiber preparation

The MWCNT-HNO_3_-β-CD, MWCNT-HNO_3_-γ-CD, MWCNT-H_2_O_2_-β-CD, and MWCNT-H_2_O_2_-γ-CD-based coatings were obtained following an already published procedure [9, 14]. Three fibers were prepared and tested for each material. For characterization, see ESM.

### Optimization and validation of the SPME–GC–MS method

A 2^3^ full factorial design followed by a desirability function approach was carried out to investigate the effects of desorption time, extraction temperature, and extraction time using the MWCNT-H_2_O_2_-γ-CD fiber. The optimized procedure is briefly reported: 19.5 mL of sample was extracted in DI-SPME mode at 40 °C for 80 min. The fiber was desorbed in the GC–MS injector for 4 min at 270 °C. The detection mode was time-scheduled selected ion monitoring (SIM). Finally, the SPME–GC–MS method was validated according to EURACHEM guidelines [24]. More details are in ESM.

### Nuclear magnetic resonance (NMR)

^1^H-NMR titrations were carried out by adding twelve aliquots (up to 400 μL) of a 10^−2^ M solution of γ-CD in DMSO [D_6_] to a 10^−3^ M solution (500 μL) of pyrene prepared in a 5 mm NMR tube. The chemical shift variation of the PAH signals was monitored after γ-CD addition and compared with those of the free species (see ESM).

### Fluorescence measurements

Fluorescence measurements were performed using Py as model compound: briefly, 10 μM Py solution was titrated by adding consecutive aliquots of an aqueous γ-CD solution (500 μM) reaching a host:guest ratio of 21.75. Fluorescence was recorded using an excitation *λ* of 385 nm, while emission was recorded in the 345–440 nm range (other information in ESM).

### Solid state analysis

Crystals suitable for X-ray diffraction were obtained as follows: γ-CD and Py in 1:1 molar ratio were dissolved in water and methanol, respectively, and the resulting solutions were mixed and stirred for 2 h at room temperature. Subsequently, 200 mL of acetonitrile was added, and the mixture was evaporated until a volume of 20 mL. Finally, it was stirred for two days at 70 °C, and the colorless prismatic crystals formed upon cooling were analyzed. Additional information is in ESM.

### Real sample analysis

Four alpine snow samples were collected in the ski area Dolomiti di Brenta at an altitude over 2000 m in December 2022 (coordinates in ESM): snow was manually sampled using solvent-rinsed glass bottles (100 mL volume), after removal of the first 10 cm of the snowpack to prevent contamination.

## Results and discussion

MWCNTs and CDs were the individual components chosen for the development of the hybrid nanocomposite material, considering both the commercial availability and the ability to strongly interact with the target analytes. Taking into account that the major drawbacks in paleoclimatic studies rely on both the achievement of very low detection/quantitation limits and reduced sample volume availability, the presence of synergistic effects between a nanostructured material characterized by high surface and the binding capability of the supramolecular receptor could play a key role in increasing the SPME extraction capabilities.

### Characterization of the MWCNT nanocomposite materials

To increase the number of carboxylic groups and facilitate interactions with CDs, an oxidation procedure using both HNO_3_ and H_2_O_2_ was applied using COOH-MWCNTs as starting material. COOH-MWCNTs oxidized with HNO_3_ resulted in the increase of carboxylic groups onto the surface (0.34, 1.45, and 0.94 mmol/g for COOH-MWCNTs, MWCNT-HNO_3_, and MWCNT-H_2_O_2_, respectively), as also confirmed by the higher *ζ* potential (− 7.9, − 31.1, and − 12.5 mV for COOH-MWCNTs, MWCNT-HNO_3_, and MWCNT-H_2_O_2_, respectively). Treatment with H_2_O_2_ was also successful in oxidizing the nanotubes, but to a lesser extent.

All obtained materials were characterized by FT-IR and TGA, observing characteristic peaks related to cyclodextrins on both HNO_3_ and H_2_O_2_-treated MWCNTs (Fig. S1, Table S1) and superior thermal stability compared to commercial COOH-MWCNTs (Table S2). The amount of CD in the final material was evaluated by TGA, β-CD accounting for 3.2% and 4.5% of the total weight after treatment with HNO_3_ and H_2_O_2_, respectively. A similar trend was observed with γ-cyclodextrin, which was present at 4.7% and 5.5% on the COOH-MWCNTs after HNO_3_ and H_2_O_2_ treatments, respectively. CDs are probably linked to the MWCNTs *via* polar-polar interactions, hydrogen bonds, and Van der Waals forces. With respect to the starting material, the amount of CDs increases with the COOH number. Unfortunately, the HNO_3_ treatment was found to lead to a partial deterioration of the MWCNTs as already observed in [22], resulting in a deterioration of the coating.

**Fig. 1:**
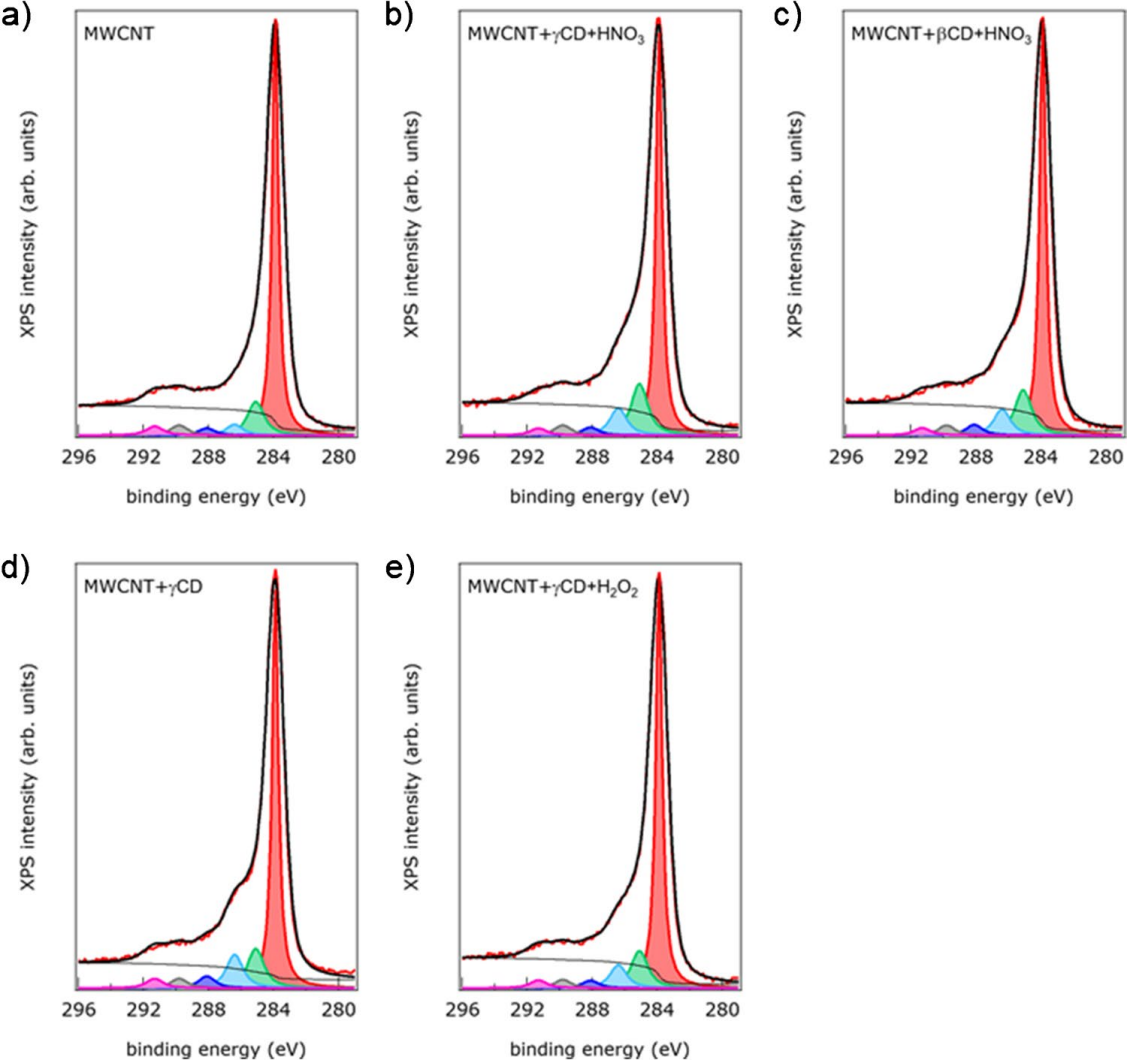
C1s XPS spectra (red line) of the sample MWCNTs as synthesized (**a**), before and after oxidation with HNO_3_ and functionalization with γ -CD (**b**) and with β-CD (**c**), after functionalization with γ-CD (**d**) and after oxidation with H_2_O_2_ and functionalization with γ-CD (**e**). The fitting curve (thick black line), the background (thin black line), and the fitting components, representing C–C bonds (red), defects and C bound to OH groups (green), C atoms bound to one, two, and three O atoms (light blue, dark blue, and grey, respectively), and π-π* transition losses (magenta) are also shown

XPS was used to gain insight into the surface chemistry of the developed materials. Figure 1 shows the C1s spectra of the samples before and after the different functionalization with CDs.

The spectra appear asymmetric on the high binding energies’ side, due to the presence of C atoms with different bonds. All samples showed a dominant peak at 284 eV attributable to graphitic bonds [22, 25]. The second component at 285.1 eV is ascribed to defects and C bound to OH groups [22, 25]. The three components at 286.4 eV, 288.1 eV, and 288.8 eV are attributed to C atoms bound to one, two, and three O atoms, respectively [22, 25]. The component at 291.3 eV is ascribed to the π-π* transition losses [22]. C-O bonds are expected to have a binding energy for C1s core levels between 286 and 287 eV, overlapping with the component attributed to C atoms bound to N via C-N bonds [26]. C bonded to two O atoms is expected to have a C1s binding energy of approximately 288 eV, overlapping the peak attributed to N–C = O bonds [27]. Since the intensity of the N1s signal was below the noise level both before and after the treatments, the observed signals can only be attributed to C-O bonds. Table S3 reports the relative intensity of the peaks ascribed to the different C species, obtained by fitting the spectra in Fig. 1. In general, a decrease in the concentration of graphitic carbon and an increase of the concentration of other C-related species were observed on the functionalized materials compared to bare COOH-MWCNTs. On both the γ- and β-CD nanotubes treated with HNO_3_, a slightly higher concentration of C atoms bound to OH species was detected compared to the treatment with H_2_O_2_.

The O1s spectra of the samples before and after the different functionalization treatments are reported in Fig. S2. The spectra were fitted using a Voigt-shaped peak at 532.8 eV binding energy attributed to carboxyl groups and one at 531.2 eV attributed to hydroxyl groups [22]. The relative intensities of the different peaks obtained from the fitting are summarized in Table S4. The bare COOH-MWCNT sample shows relatively low intensity of the O1s overall signal with peaks at 532.8 eV and at 531.2 eV, which dominate the spectrum. After functionalization, the intensity of the O1s component at 532.8 eV increased significantly.

In conclusion, XPS data showed that the functionalization induced an increase in the concentration of surface C species bound to H and O and of surface O species bound to C and H.

### Performance of the nanocomposite-based SPME fiber coatings

**Fig. 2:**
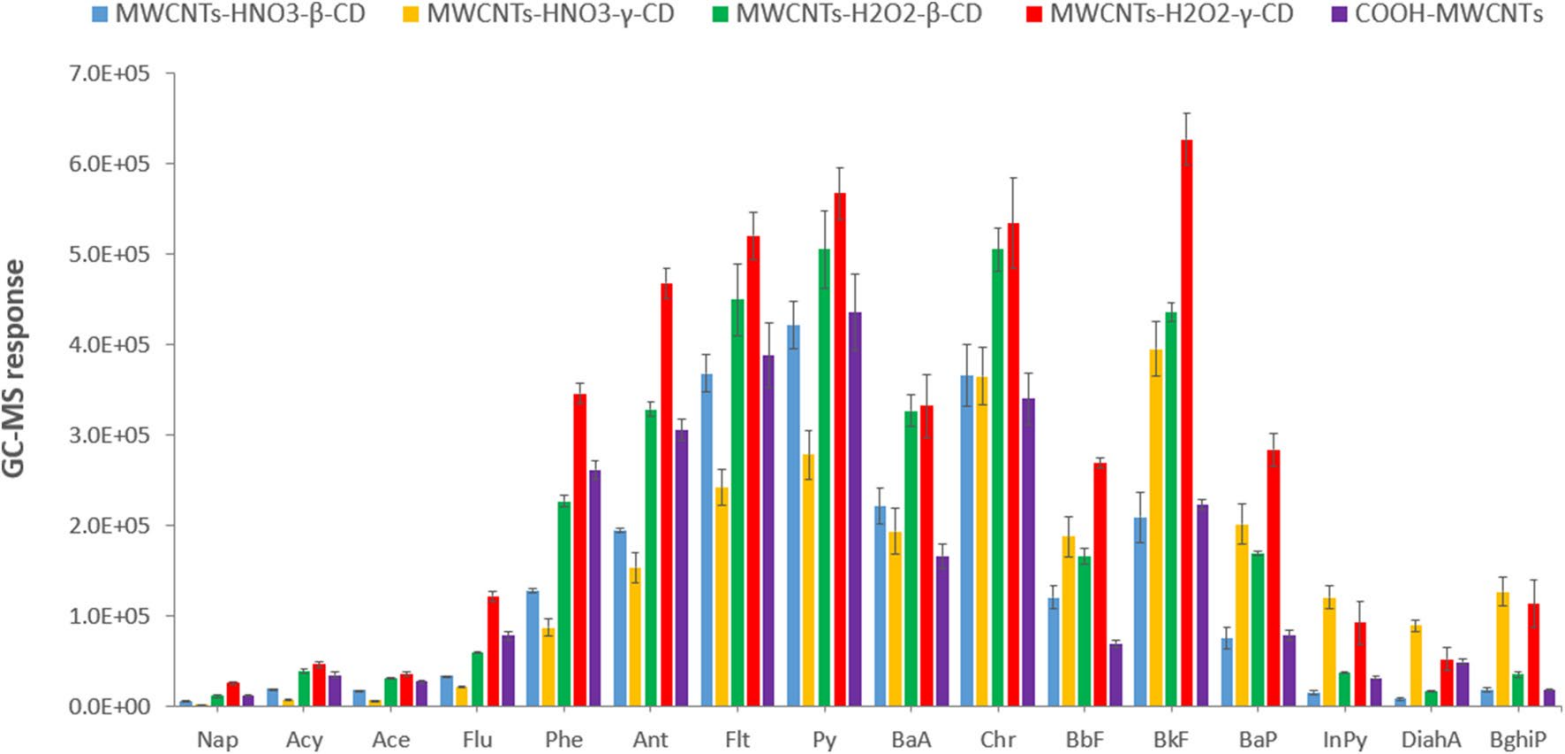
Comparison of the GC–MS responses obtained by extracting the 16 US-EPA using the developed fiber and commercially available COOH-MWCNT coatings (extraction time: 45 min, extraction temperature: 50 °C; desorption temperature: 270 °C, desorption time, 2 min) (*n* = 3)

To select the most suitable coating for PAH extraction, the performance of the MWCNT-HNO_3_-β-CD, MWCNT-HNO_3_-γ-CD, MWCNT-H_2_O_2_-β-CD, MWCNT-H_2_O_2_-γ-CD, and COOH-MWCNT fibers were compared in terms of GC-MS responses (Fig. 2).

As shown in Fig. 2, a significant decrease in terms of GC-MS response was observed for commercially available COOH-MWCNTs after a reduced number of extraction-desorption cycles (up to 7-fold lower responses after 9 cycles). This behavior could be ascribed to the decarboxylation of oxidized COOH-MWCNTs that occurs in the GC injection port [28]. Similarly, CDs used alone were not stable at the high temperatures required for the complete desorption of the heaviest PAHs. By contrast, the coatings based on the developed nanocomposite materials proved to be stable over 270 °C, with negligible weight losses of 7% and 1% for β-CD and γ-CD-based materials, respectively (Table S1).

The best extraction performance was achieved using the hydrogen peroxide-based treatment. These findings can be explained considering that strong oxidizing agents such as nitric acid can produce exfoliation and etching of the nanotubes; on the contrary, a lower degree of damage was obtained when H_2_O_2_ treatment was applied [22]. These results are in agreement with the previously reported *ζ* potential measurements.

As for the extraction performances of MWCNT-H_2_O_2_-γ-CD and MWCNT-H_2_O_2_-β-CD, better results were obtained using the nanocomposite functionalized with γ-CD, thus assuming that this receptor is able to complex PAHs more effectively. These findings are consistent with those reported by Belenguer-Sapiña et al. [17], who developed silica structures containing bound CDs for the determination of phenolic compounds in air and PAHs in water samples. As already demonstrated, the complementarity of the cavity size with the steric hindrance of the analytes plays a key role in promoting their extraction from water: β-CD and γ-CD have cavity diameters of 0.78 and 0.95 nm, respectively, while PAHs have volumes in the 0.8–0.9 nm range. Therefore, medium and high-molecular weight PAHs are not fully included inside the hydrophobic cavity of β-CD, resulting in weaker host–guest interactions. By contrast, PAHs fit the γ-CD cavity, which also has greater flexibility to host analytes with the most suitable orientation, leading to strong complexes via Van der Waals and π- π interactions.

**Fig. 3:**
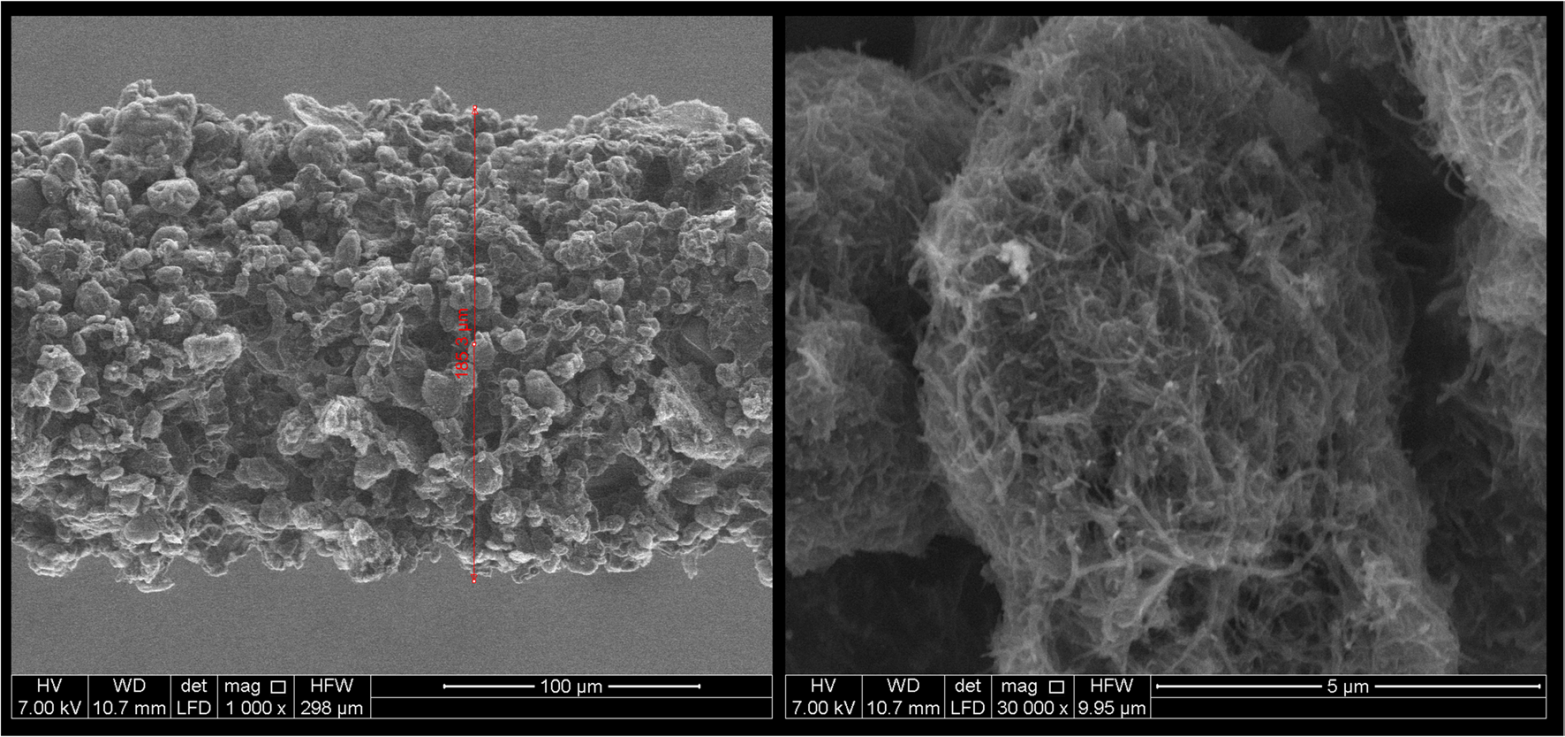
ESEM images of a MWCNT-H_2_O_2_-γ-CD-coated fiber; magnification: 1000 × (left) and 30,000 × (right)

As shown in Fig. 2, up to 6-fold higher GC–MS responses were obtained when the MWCNT-H_2_O_2_-γ-CD-coated fiber was used compared to those achieved using COOH-MWCNT coating. The morphological characterization of the coating was performed by environmental scanning electron microscopy (ESEM) resulting in a uniform distribution of the nanocomposite with an average thickness of 42 ± 2 μm (*n* = 4) (Fig. 3). In addition, this fiber was also characterized by greater thermal stability, thus being used for the subsequent optimization and validation steps.

### Optimization of the SPME conditions

Extraction conditions in terms of desorption time (*x*_1_), extraction temperature (*x*_2_), and extraction time (*x*_3_) were optimized as main factors by means of a CCF design. Based on previous knowledge, the following experimental domains were investigated: (i) *x*_1_ in the 3–5 min range to obtain the complete desorption of the analytes, thus avoiding carryover effect; (ii) *x*_2_ in the 40–60 °C range to maintain stable temperatures inside the autosampler heater and prevent the desorption of the most volatile compounds from the SPME coating; (iii) based on the results reported in our previous study [9], 40 min was considered as minimum extraction time to obtain the absorption of the analytes onto the coating and 80 as maximum time limit. The desorption temperature was kept at 270 °C that was considered an adequate temperature for complete desorption of the analytes while avoiding coating degradation.

For each analyte, the significance of linear, quadratic, and interaction effects was evaluated using a forward search step-wise variable algorithm (*p* to remove 0.05). The obtained models are reported in Table S5.

As reported, *x*_1_ has no significant effect for all the PAHs except Chr, which presents a negative quadratic effect; therefore, the maximum response is associated with an intermediate desorption time. As for *x*_2_, a different effect is present, depending on the molecular weight of the analytes: low-molecular weight compounds have a negative coefficient, being these compounds partially desorbed from coating at high temperatures, whereas the extraction of medium-sized PAHs is favored when temperature is increased up to 60 °C. This effect could be explained considering that temperature increases the overall extraction kinetics, thus enhancing the diffusion coefficients of the analytes. As for *x*_3_, it positively affects the extraction of PAHs with molecular weight higher than Flu; therefore, prolonged extraction times are required to promote the adsorption of these analytes onto the nanocomposite coating.

To obtain the overall conditions for the simultaneous extraction of the 16 PAHs, the multicriteria method of the desirability functions was applied [29, 30]: the optimal conditions were *x*_1_: 4 min, *x*_2_: 40 °C, and *x*_3_: 80 min, achieving a global desirability *D* = 0.81.

### Method validation

Method validation was performed using the developed nanocomposite MWCNT-H_2_O_2_-γ-CD coating under the optimized extraction conditions. Excellent results were obtained in terms of detection and quantitation limits always below 0.7 and 2.3 ng/L, respectively, thus proving the suitability of the SPME–GC–MS method for the determination of PAHs at ultratrace levels (Table 1). For most of the investigated PAHs, the achieved results were up to 4 times lower than those obtained in a previous study, based on the use of helical MWCNTs [9], strengthening the pivotal role of CD in interacting with the investigated analytes. The synergic effect of MWCNTs and γ-CD was further confirmed by the comparison with literature data: as reported in Table S6, the use of the nanocomposite coating produced a strong decrease in the detection limits, thus allowing the analysis of pollutants at very low concentration levels. In addition, compared to previously published studies mainly based on SPE or DSPE, SPME resulted in an automatable procedure based on thermal desorption of analytes and reduced sample handling.

**Table 1:** LODs, LOQs, linearity range (*y* = *b*0 + *b*1*x*) and regression coefficients for the 16 US-EPA PAHs

Compound	LOD	LOQ	Regression coefficients	
	ng/L			
Nap	0.7	2.3	-	0.015 (± 0.001)
Acy	0.7	2.2	-	0.062 (± 0.001)
Ace	0.4	1.4	-	0.058 (± 0.001)
Flu	0.2	0.8	-	0.14 (± 0.01)
Phe	0.1	0.3	-	0.036 (± 0.009)
Ant	0.2	0.7	-	0.042 (± 0.002)
Flt	0.1	0.2	-	0.41 (± 0.01)
Py	0.1	0.3	1.2 (± 0.4)	0.44 (± 0.02)
BaA	0.2	0.6	-	0.151 (± 0.005)
Chr	0.1	0.5	-	0.64 (± 0.01)
BbF	0.2	0.8	-	0.175 (± 0.004)
BkF	0.3	0.9	-	0.86 (± 0.02)
BaP	0.3	1.0	-	0.36 (± 0.01)
InPy	0.2	0.6	-	0.30 (± 0.01)
DiahA	0.3	0.9	-	0.30 (± 0.01)
BghiP	0.3	0.9	0.5 (± 0.1)	0.35 (± 0.01)

(-): not significant

Method linearity was assessed in the LOQ–30 ng/L range by applying Mandel’s fitting test. Good precision was demonstrated in terms of both repeatability and intermediate precision, with RSD lower than 21% (Table S7).

As for the intermediate precision, ANOVA showed that mean values were not significantly different among the 3 days (*p* > 0.05). Trueness was assessed for 3 different concentration levels (3, 10, and 20 ng/L), obtaining RR in the 88(± 2)–119.8(± 0.4)% range (Table S8).

As for batch-to-batch repeatability, ANOVA demonstrated the absence of significant differences among the responses (*p* > 0.05), assessing good repeatability of the coating procedure. Selectivity was evaluated by analyzing snow samples and verifying the absence of any interfering peaks around the analyte retention times. Finally, the performances of the MWCNT-H_2_O_2_-γ-CD-coated SPME fibers were evaluated in terms of enrichment factors (EFs), with values in the 3770 (± 260)–113,300 (± 3100) range. EFs up to 3 times higher than those reported in our previous study [9] were obtained for medium- and high-molecular weight compounds. These findings can be ascribed to the synergistic effect related both to the presence of γ-CD on the surface of the nanotubes, allowing the complexation of the analytes within the macromolecular receptors, and to the π-π interactions between PAHs and nanotubes. In addition, stronger interactions can be built between PAHs and the inner CD cavity since medium- and high-molecular weight PAHs are more hydrophobic than lighter ones.

The reusability of the developed coating was also evaluated: more than 50 extractions could be performed without significant changes in the GC–MS responses. Although this is not an impressive result if compared to the lifetime of some commercially available coatings, the fiber reusability was found to be better than that reported for most of the in-lab developed materials reported in Table S6.

Finally, it can be stated that the synthesis of the nanocomposite coating does not require a laborious approach, resulting comparable or simpler than other CD-based materials, mostly based on functionalized microporous silica or graphene oxide magnetic particles.

### Assessment of the γ -CD–PAH interactions

Preliminary solution complexation studies were performed to clarify the interactions between PAHs and the nanotubes functionalized with γ-CD. However, it is worth noting that these studies cannot be considered representative of the actual interactions operating in the composite material since in solution the solvation of the CD cavity and PAHs usually adversely affects the complexation process. Previous studies have shown that the forms present in solution are the result of a fast equilibrium between free and complexed species, with the stoichiometry of the complex mainly driven by the steric complementarity between the host and the guest with different stoichiometries (1:1, 1:2, and 2:1) occurring simultaneously [21].

NMR spectroscopy was used as an investigation tool: Py was selected as the representative guest molecule for the PAH family. In principle, its prolate shape and size would favor the formation of 1:1 or 2:1 host-guest adducts thanks to the inclusion of longitudinal pyrene rings in one or two hydrophobic cyclodextrin cavities, respectively. However, a different arrangement of the guest molecule in the complex in which either two CD units are sandwiched over a flat PAH molecule or two stacked pyrene molecules are simultaneously deeply engulfed in the CD cavity cannot be excluded [31, 32].

DMSO-d_6_ was used as solvent since it is the only one capable of dissolving γ-CD and Py in appreciable amounts for NMR measurements. Upon adding incremental amounts of γ-CD solution, a downfield shifting of all Py resonances was observed, together with broadening and signals splitting (Fig. S3). These findings showed that host-guest complexation process is close to the slow-exchange conditions: unfortunately, this kinetic condition prevented the calculation of the binding constant.

The host-guest complex formation in solution was also investigated by fluorescence spectroscopy, since an increase in the intensity of emission bands is expected during CD@Py complexation [33]. Titration of Py using γ-CD showed an enhancement of the emission by increasing the host concentration. The maximum emission intensity was then plotted against the γ-CD concentration for each titration point, and complexation models suggested the formation of 2:1 host-guest complex with a log(*β*2) of 7.30 ± 0.09 (Fig. S4).

### Solid state investigations

Solid state investigations on the system γ-cyclodextrin/PAHs were carried out using the Cambridge Structural Database [34] which yielded the structures: [bis(β-cyclodextrin) octanol clathrate pyrene hydrate] (refcode PUKPIU) and [bis(β-cyclodextrin) tris(cyclohexanol) clathrate pyrene hydrate] (refcode PUKPOA) [35]. In both cases, the alcohol (in blue) forms a host-guest complex with the macrocycle, while Py (in green) is sandwiched between two β-cyclodextrins by means of dispersion interactions (Fig. S5).

Inspired by these results, crystallization experiments with γ-cyclodextrin and pyrene were set up and the crystals obtained were analyzed through X-ray diffraction. Albeit it was not possible to fully refine the structure, the skeleton of the cyclodextrin could be clearly modelled (Fig S6), whereas some significant, residual electron density (green mesh) was sandwiched between two cyclodextrins, similarly to the position of pyrene in the examples of the β-cyclodextrin clathrates previously discussed.

### Real sample analysis

The results obtained analyzing the snow samples collected in the ski area Dolomiti di Brenta are reported in Table 2. PAHs were present at very low concentration levels, and in particular, naphthalene, phenanthrene, fluoranthene, and pyrene were detected in all the samples analyzed, while among the high molecular weight compounds a greater number of 4 and 5 rings were found in sample 4. According to previous results [14], naphthalene was the most abundant compound accounting for 29.9–61.4% of the ∑16PAHs, whereas 3- and 4-ring PAHs were in the 38.6–61.3% range. The presence of PAHs in alpine snow is not surprising and has already been explained considering that, during snowfall, PAHs absorbed on particulate matter can be removed from the atmosphere and entrapped into snowflakes.

**Table 2:** Concentration (ng/L) of the 16 US-EPA PAHs monitored in the alpine snow samples

	Sample 1	Sample 2	Sample 3	Sample 4
Naphthalene	9.81 ± 0.34	5.93 ± 0.22	6.76 ± 0.47	10.18 ± 0.65
Acenaphthylene	n.q	n.d	n.q	2.30 ± 0.04
Acenaphthene	1.40 ± 0.07	n.d	n.d	n.q
Fluorene	1.73 ± 0.07	n.q	1.15 ± 0.05	2.22 ± 0.10
Phenanthrene	3.42 ± 0.11	0.64 ± 0.06	1.15 ± 0.25	3.46 ± 0.47
Anthracene	n.d	n.d	n.d	n.d
Fluoranthene	2.05 ± 0.09	2.64 ± 0.12	1.08 ± 0.05	4.63 ± 0.21
Pyrene	3.42 ± 0.11	1.94 ± 0.16	0.87 ± 0.03	6.24 ± 0.18
Benzo[a]anthracene	n.d	n.d	n.q	0.85 ± 0.06
Chrysene	0.75 ± 0.03	n.d	n.q	1.13 ± 0.05
Benzo[b]fluoranthene	n.q	n.d	n.d	1.18 ± 0.04
Benzo[k]fluoranthene	n.d	n.d	n.d	n.d
Benzo[a]pyrene	n.q	n.d	n.d	n.q
Indeno[1,2,3-c,d]pyrene	0.67 ± 0.03	n.d	n.d	0.73 ± 0.02
Dibenzo[a,h]anthracene	n.q	n.d	n.d	1.05 ± 0.08
Benzo[g,h,i]perylene	n.q	n.d	n.d	n.q

*n.d.* not detected (< LOD), *n.q.* not quantitated (< LOQ)

## Conclusions

The combined use of MWCNTs and γ-CD enabled the development of an effective and low-cost SPME–GC–MS method for PAH extraction at ultratrace levels, paving the way for a better evaluation of pollutant sources and contamination patterns in paleoclimatic studies. Additional figure of merit was the compliance with the principles of green analytical chemistry during both the synthesis of the material and the analytical workflow in terms of reduced consumption of organic solvents, no waste production, material reusability, automation and miniaturization of the extraction procedure, and low sample consumption. Therefore, the proposed procedure can be considered a good alternative to other approaches commonly used for PAH determination.

Future investigations will be performed to assess the extraction capability of the developed material towards different classes of analytes in matrices of environmental and food concern.

## Supplementary information

Below is the link to the electronic supplementary material.

Supplementary file1 (DOCX 2.34 MB)

## Data Availability

Data are available from the corresponding author upon reasonable request.
