# Supplementary material for: A novel multiwalled carbon nanotube–cyclodextrin nanocomposite for solid-phase microextraction–gas chromatography–mass spectrometry determination of polycyclic aromatic hydrocarbons in snow samples
Source: Mikrochim Acta. 2023 May 12;190(6):212. doi: 10.1007/s00604-023-05799-8 (PMC10181969; doi:10.1007/s00604-023-05799-8)
Supplement: Supplementary file 1 — Supplementary file1 (DOCX 2.34 MB) [file 604_2023_5799_MOESM1_ESM.docx]

**SUPPLEMENTARY INFORMATION**

**A novel multiwalled carbon nanotube–cyclodextrin nanocomposite for Solid-Phase Microextraction–Gas Chromatography–Mass Spectrometry determination of polycyclic aromatic hydrocarbons in snow samples**

N. Riboni^1*^, F. Bianchi^1,2^*, M. Scaccaglia^1^, F. Bisceglie^1^, A. Secchi^1^, C. Massera^1^, P. Luches^3^, M. Careri^1^

*^1^University of Parma, Department of Chemistry, Life Sciences and Environmental Sustainability, Parco Area delle Scienze 17/A, 43124 Parma, Italy*

*^2^University of Parma, Center for Energy and Environment (CIDEA), Parco Area delle Scienze 42, 43124, Parma, Italy*

*^3^Nanoscience Institute, CNR, via G. Campi 213/A, 41125 Modena, Italy*

**Corresponding Authors**

*Nicolò Riboni: e-mail: nicolo.riboni@unipr.it. Phone: +39 0521 905448.

*Federica Bianchi: e-mail: federica.bianchi@unipr.it. Phone: +39 0521 905446.

**ORCID**

Nicolò Riboni: 0000-0002-0789-3812

Federica Bianchi: 0000-0001-7880-5624

Mirco Scaccaglia: 0000-0001-9232-8772

Franco Bisceglie: 0000-0001-9165-0356

Andrea Secchi: 0000-0003-4045-961x

Chiara Massera: 0000-0003-0230-1707

Paola Luches: 0000-0003-1310-5357

Maria Careri: 0000-0002-4558-5001

The experimental section is reported herein. Analytical and spectral characterizations of the developed coating like FT-IR, XPS spectra and TGA and ζ-potential data are provided in this section in order to better support the characterization of the developed materials. Supporting figures related to NMR, fluorescence spectroscopy and solid-state analysis are also present. Tables related to method optimization and validation are also reported.

**Chemicals and Materials**

EPA 525 PAH Mix A including naphthalene (Nap), acenaphthylene (Acy), acenaphthene (Ace), fluorene (Flu), phenanthrene (Phe), anthracene (Ant), fluoranthene (Flt), pyrene (Py), benzo[a]anthracene (BaA), chrysene (Chr), benzo[b]fluoranthene (BbF), benzo[k]fluoranthene (BkF), benzo[a]pyrene (BaP), indeno[1,2,3-c,d]pyrene (InPy), dibenzo[a,h]anthracene (DiahA), benzo[g,h,i]perylene (BghiP) (each at 500 µg/mL in dichloromethane), and PAH-Dx–Mix 16 containing Nap-d8, Acy-d8, Ace-d10, Flu-d10, Phe-d10, Ant-d10, Flt-d12, Py-d10, BaA-d12, Chr-d12, BbF-d12, BkF-d12, BaP-d12, InPy-d12, DiahA-d14, BghiP-d12 (each at 10 mg/L in acetonitrile) to be used as internal standards (ISs) were purchased from NEOCHEMA GmbH (Bodenheim, Germany).

One cm length SPME bare fused-silica fibers were purchased from Merck (Bellefonte, PA, USA). Duralco 4460 epoxy glue was provided by Cotronics Corp. (Brooklyn, NY, USA)

β-cyclodextrin and γ-cyclodextrin were purchased from Sigma Aldrich.

Graphitized multi-walled-COOH carbon nanotubes (COOH-MWCNTs, ⌀ 50 nm and 10–20 μm length), were purchased from Cheap Tubes Inc. (Cambridgeport, Massachusetts, USA).

**COOH-MWCNTs functionalization and characterization**

Treatment 1 with HNO_3_: 500 mg of the COOH-MWCNTs were dispersed in 20 mL of the nitric acid (65 wt%) in a 50 mL round bottom flask and stirred at room temperature for 72 hours. The resulting dispersion was filtered on a frit funnel. The solid (MWCNT-HNO_3_) was washed with water up to pH = 7 and the sample was dried in vacuum.

Treatment 2 with H_2_O_2_: 500 mg of the COOH-MWCNTs were dispersed in 20 mL of ammonium hydroxide (25 wt%) and 20 mL of hydrogen peroxide (30%) in a 100 mL round bottom flask and reflexed under stirring for 5 hours. The resulting dispersion was filtered on a frit funnel. The solid (MWCNT-H_2_O_2_) was washed with water up to pH = 7 and the sample was dried in vacuum.

The further functionalization with the two different cyclodextrins were conducted treating 100 mg of the oxidized MWCNTs with 200 μL of hydrazine and 800 mg of each cyclodextrin in 20 mL of water with 1 mL of ammonium hydroxide (30%) at 95 °C for 3 hours. Reduced MWCNTs were obtained with the same treatment, without the addiction of any cyclodextrin.

The total acidic sites concentration was determined with a reverse acid-base titration: 100 mg of oxidized CNTs were sonicated for 30 min in 100 mL of 10^-2^ M NaOH. After the sonication, mixtures were left to equilibrate overnight. The supernatant was titrated with a 10 ^-2^ M HCl solution to determine the excess NaOH in the solution and the concentration of the carboxylates on CNTs.

In order to analyse the effect of the appied treatments on the COOH-MWCNTs and compare them with the material commercially available, different analytical techniques were used. Fourier Transform InfraRed spectroscopy (FT-IR) measurements were recorded on Perkin Elmer’s Spectrum Two in the 2000-200 cm^-1^ range. The changes in zeta potentials were measured in deionized water at pH 7.4 using a zeta potential analyser (Malvern Zetasizer Nano ZSP).XPS measurements were performed in an ultra-high vacuum chamber equipped with a hemispherical electron analyzer (Omicron Nanotechnology, Germany) and a double anode x-ray source (Leybold-Heraeus GmbH, Germany). For the XPS measurements each sample was distributed uniformly on top of a piece of a double-sided adhesive carbon tape attached to the sample holder. Wide scan spectra were acquired using a pass energy of 50 eV, while for narrow scan spectra a pass energy of 30 eV was used. Thermogravimetric analysis (TGA) was performed using a TGA 7 instrument (Perkin-Elmer, Walthan, MA) over the temperature range 40-400°C (heating rate: 10°C/min) under inert (N_2_) atmosphere.

**Fiber characterization**

Coating thickness and surface morphology of the developed fibers were investigated by scanning electron microscopy (SEM) using a Quanta ™ 250 FEG instrument (Thermo Fisher Scientific Waltham, Massachusetts, US).

Fiber bleeding was investigated by desorbing the fibers in the GC injection port for 2 min at 270°C. Fiber-to-fiber and batch-to-batch repeatability were evaluated by using 3 fibers always performing 3 replicated measurements per fiber. Solutions at 20 ng/L were always analyzed.

**GC-MS analysis**

GC-MS analyses were performed using a HP 6890 Series Plus gas chromatograph (Agilent Technologies, Palo Alto, CA) equipped with a MSD 5973 mass spectrometer (Agilent Technologies). Helium was used as carrier gas at a constant flow rate of 1.2 mL/min; the GC injector was operated in splitless mode at 270°C for each material whereas chromatographic separation was performed on a Rxi-17Sil MS capillary column (30 m × 0.25 mm i.d., 0.25 μm film thickness; Restek, Bellafonte, USA), using the following temperature programme: 70 °C, held for 0.50 min, 15 °C/min to 290 °C, held for 4.00 min. The mass spectrometer was equipped with an electron ionization ion source (70 eV). Transfer line and ion source were maintained at 280°C and 150°C, respectively. GC-MS analyses were carried out under selected ion monitoring (SIM) conditions setting a dwell time of 30 ms. The current of the following ions was recorded: *m/z* **128**, 127 and 102 for Nap from 1 to 5.0 min; *m/z* **152**, 151 and 76 for Acy and *m/z* 154, **153** and 76 for Ace from 5.0 to 7.0 min; *m/z* **166**, 165 and 139 for Flu from 7.0 to 8.5 min; *m/z* **178**, 176 and 152 for Phe and Ant from 8.5 to 10.0 min; *m/z* **202**, 200 and 101 for Py and Flt from 10.5 to 12.0 min; *m/z* **228**, 226 and 113 for BaA and Chr from 12.0 to 14.0 min; *m/z* 253, **252** and 126 for BbF, BkF and BaP from 14.0 to 16.0 min; *m/z* 279, **278**, 276, 277, 139 and 138 for BghiP, DiahA and InPy from 16.0 to 19.2 min (in bold the ions used for quantitation purposes). As for the ISs, the same fragmentations were considered, by correcting the respective *m/z* values for the number of deuterated atoms. An electron multiplier voltage of 2400 V was applied. Signal acquisition and data handling were performed using the HP Chemstation (Agilent Technologies).

**MWCNTs-based coatings performance**

Prior to use, the MWCNTs-HNO_3_-β-CD, MWCNTs-HNO_3_-γ-CD, MWCNTs-H_2_O_2_-β-CD and MWCNTs-H_2_O_2_-γ-CD fibers were conditioned in the GC injection port for 1 h under a helium flow. All the analyses were performed using a PAL COMBI-xt autosampler (CTC Analytics AG, Zwingen, Switzerland) operating in direct immersion mode using 19.5 mL of aqueous sample in 20 mL glass vials. The performance of the four developed coatings was tested by analyzing not contaminated water spiked with the 16 PAHs at the concentration of 20 ng/L. After incubation at 50°C for 5 minutes, SPME extraction was carried out at 50°C for 45 min, under an agitation speed of 250 rpm. The extraction performances of the coatings were evaluated in terms of GC-MS responses.

**Method optimization**

All the experiments were carried out using blank tap water spiked with each analyte at 50 ng/L. The experimental domain was explored in the 3-5 min, 40-60°C and in the 40-80 min range in terms of desorption time, extraction temperature and time, respectively. Four replicates at the center of the experimental domain were used to evaluate the experimental error. The presence of relevant quadratic effects was assessed by running a F-test by comparing the experimental and calculated responses at the center of the experimental domain.

The significance of the main factors and their interactions was evaluated. The regression models were obtained by a forward search stepwise variable algorithm (*p* to remove 0.05) by using the statistical package SPSS Statistics v.23.0 (IBM, Milan, Italy). Finally, the multicriteria method of the desirability functions [1–3] was used to search for the optimal extraction conditions of the 16 PAHs.

**Method validation**

. Not contaminated tap water was used as blank matrix. Detection (y_D_) and quantitation (y_Q_) limits were calculated as signals based on the mean blank (x_b_) and the standard deviation of blank responses (s_b_) as follows: y_D_=x_b_+3 s_b_ and y_Q_ = x_b_ + 10 s_b_. The value of x_b_ and s_b_ were calculated performing ten blank measurements. Detection and quantitation limits (LOD and LOQ, respectively) were obtained by projection of the corresponding signals y_D_ and y_Q_ through a calibration plot y = f(x) onto the concentration axis. Linearity was established on six concentration levels, three replicated measurements for each level, in the LOQ- 30 ng/L range. Homoscedasticity was verified by applying the Bartlett test. Lack of fit and Mandel's fitting test were also performed to assess the goodness of fit and linearity. The significance of the intercept (significance level 5%) was tested by running a Student's t-test. Precision in terms of repeatability and intermediate precision was calculated at three concentration levels (LOQ, 15 and 30 ng/L): 6 replicated measurements were always performed. Intermediate precision was estimated over 3 days using three different fibers, verifying the homoscedasticity of data and performing the analysis of variance (ANOVA) at the confidence level of 95%. Trueness was calculated as recovery rate (RR%) according to the following equation: RR%=c_1_/c_2_∙100 where c_1_ is the measured concentration and c_2_ is the concentration obtained by spiking the blank sample. Recovery rate values were assessed by performing ten replicated measurements per level at the LOQ, 10 and 20 ng/L for all the analytes. Selectivity was evaluated by analyzing snow samples and verifying the absence of interfering compounds.

Finally, enrichment factors (EFs) were calculated by spiking the snow with PAHs at the concentration of 10 ng/L (n=3). The ratio of the concentration of the PAHs extracted onto the fiber coating to that of the analytes in the standard mixture, i.e. using the ratio of the chromatographic peak area of each analyte after SPME compared to that obtained by the direct injection of standard solutions was used to calculated EF values [4,5].

**NMR Measurements**

^1^H-NMR spectra were recorded on a Bruker Avance III instrument operating at 400 MHz. Chemical shifts are expressed in ppm (δ) using the residual solvent signal as an internal reference (2.51 ppm for CD_3_SOCHD_2_). The terms m, s, d, t and q represent multiplet, singlet, doublet, triplet and quadruplet, respectively.

**Fluorescence Measurements**

Spectral grade or HPLC solvents were used to prepare solutions for absorption and emission measurements. Absorption spectra were recorded with a PerkinElmer Lambda 650 spectrophotometer, while emission spectra were recorded with a FLS1000 Edinburgh Fluorometer equipped with a Xe excitation source (Edinburgh Instruments Ltd, Livingston, UK). Spectra were acquired and analyzed by Fluoracle software.

Uv-vis absorption spectra were obtained by analyzing a solution of Py at a concentration in the 2.5 -25 μM range in THF. The aim was to identify the proper excitation wavelength and investigate whether clusters are formed.

Fluorescence emission analysis was performed based on the study performed by Levine et al. [6] and Dyck et al.[7]using dual path length cuvette (material UV quartz glass Spectrosil Q, path length 1 mm). To obtain a homogeneous solution and avoid bubbles during the experiments, the cuvette was agitated and allowed to stand for 30 seconds after each addition. The experiment was repeated at the same conditions using ethanolic solution of γ-CD and Py, to investigate the effect of the solvent.

Fluorescence intensity at 372 nm, corresponding to the maximum emission intensity was recorded and complexation models fitting the titration curve were tested using HypSpec software.

**Solid state analysis**

The crystals were analyzed at 200(2) K on a Bruker D8 Venture PhotonII diffractometer (CuKα radiation λ = 1.54178 Å). The raw frame data were processed using SAINT and SADABS to yield the reflection data files [8,9]. The structures were solved by Direct Methods using the SIR2019 program [10] and refined on Fo2 by full-matrix least-squares procedures, using SHELXL-2018 [11,12] in the WinGX suite v.2014.1 [13]. The structure was solved in the tetragonal space group *P*42_1_2, with cell parameters a = b = 23.7980(7), c = 31.6880(7) and α = β = γ = 90°. V = 17946.3(12) Å^3^. Theta range for data collection: 2.322 to 74.566°. Reflections collected / unique: 292906 / 18409 [R(int) = 0.1001]. The collected data did not allow the fully refinement of the structure; the macrocycle skeleton is clearly visible, but the residual electron density could not be properly modelled into any definite molecular group (see results and discussions for details).

**Real samples**

Sampling sites were as follows: sample 1 (46° 10' 8.364" N, 10° 49' 1.416" E), sample 2 (46° 12' 59.976" N, 10° 53' 44.088" E), sample 3 (46° 14' 57.66" N, 10° 48' 53.028" E), sample 4 (46° 13' 21.9" N, 10° 50' 56.364"E).

|  | |
| --- | --- |
| COOH-MWCNTs-HNO_3_ | |
|  | |
| MWCNTs-HNO_3_-β-CD | |
|  | |
| β-CD | |
|  | |
| MWCNTs- HNO_3_ -γ-CD | |
|  |  |
| COOH-MWCNTs-H_2_O_2_ |  |
|  |  |
| MWCNTs- H_2_O_2_ -β-CD |  |
|  |  |
| MWCNTs- H_2_O_2_ -γ-CD |  |
|  |  |
| γ-CD |  |

**Fig. S1** FT-IR spectra of the developed materials

**Table S1** Selected FT-IR vibrational bands for the compounds under study

| **Functional Group** | **Wavenumber (cm^−1^)** | | | | | | | |
| --- | --- | --- | --- | --- | --- | --- | --- | --- |
|  | MWCNT-HNO_3_ | MWCNT-H_2_O_2_ | β-CD | γ-CD | MWCNT-HNO_3_-β-CD | MWCNT-HNO_3_-γ-CD | MWCNT-H_2_O_2_- β-CD | MWCNT-H_2_O_2_- γ-CD |
| ν[OH]  symmetric and  antisymmetric |  |  | 3371 | 3369 | 3371 | 3368 | 3371 | 3370 |
| ν[CH_2_] |  |  | 2929 | 2928 | 2929 | 2928 | 2929 | 2928 |
| ν[C=O] | 1631 | 1636 |  |  | 1633 | 1633 | 1633 | 1632 |
| ν[C-C] |  |  | 1152 | 1151 | 1154 |  | 1155 |  |
| ν[C-C-C] | 1073 | 1075 |  |  | 1079 | 1082 | 1079 | 1077 |
| ν[O-H]  bending vibration |  |  | 1021 | 1019 | 1030 | 1023 | 1030 | 1025 |

**Table S2** Thermal stability of the developed materials

| **Material** | **Maximum thermal stability**  **(°C)** |
| --- | --- |
| COOH-MWCNTs | 140°C |
| MWCNTs-HNO_3_-β-CD | 280°C |
| MWCNTs-HNO_3_-γ-CD | 270°C |
| MWCNTs-H_2_O_2_-β-CD | 280°C |
| MWCNTs-H_2_O_2_-γ-CD | 270°C |

**Table S3** Relative intensity of the C1s components, obtained from the fit of the spectra in Figure 1

|  | **graphitic C**  **(%)** | **def, C-OH (%)** | **C-O, C-N**  **(%)** | **-O-C=O**  **(%)** |
| --- | --- | --- | --- | --- |
| COOH-MWCNTs | 77 | 15 | 5 | 3 |
| MWCNTs-HNO_3_-β-CD | 66 | 20 | 11 | 3 |
| MWCNTs-HNO_3_-**γ**-CD | 68 | 18 | 10 | 4 |
| MWCNTs-H_2_O_2_-β-CD | 66 | 16 | 13 | 5 |
| MWCNTs-H_2_O_2_-γ-CD | 71 | 16 | 10 | 3 |


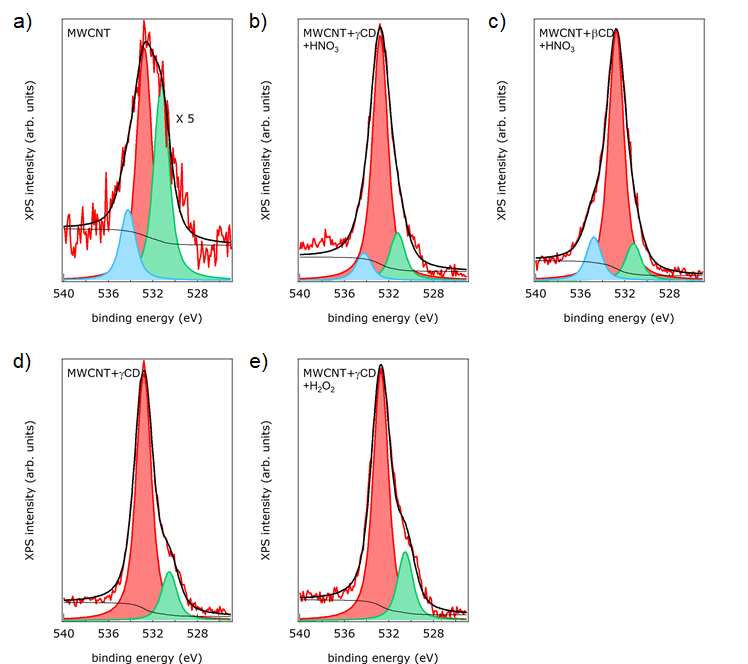


**Fig. S2.** O1s XPS spectra (red line) of the MWCNTs as synthesized (a), after oxidation with HNO3 and functionalization with γ-CD (b) and with β-CD (c), after functionalization with γ-CD (d) and after oxidation with H2O2 and functionalization with γ-CD (e). The fitting curve (thick black line), the background (thin black line) and the fitting components, representing carboxyl groups (red), hydroxyl groups (green), and physisorbed water (light blue) sample before and after different functionalization with CDs. The fitting curve (black line), the background and the fitting components are also shown

**Table S4** Relative intensity of the O1s components, obtained from the fit of the spectra in Figure S2

|  | **C-O-C, C-OH (%)** | **-OH, C=O, O-C=O (%)** |
| --- | --- | --- |
| COOH-MWCNTs | 54 | 46 |
| MWCNTs-HNO_3_-β-CD | 87 | 13 |
| MWCNTs-HNO_3_-**γ**-CD | 84 | 16 |
| MWCNTs-H2O2-β-CD | 84 | 16 |
| MWCNTs-H2O2-γ-CD | 78 | 22 |

**Table S5** Regression models and single desirability (d) for each analyte

| PAH | Regression model | d |
| --- | --- | --- |
| Nap | y = 10280 (± 640) – 1300 (± 510)x_2_ | 0.91 |
| Acy | y = 28400 (± 2400) – 13200 (± 2000)x_2_ | 0.92 |
| Ace | y = 24100 (± 1500) – 11300 (± 1200)x_2_ | 0.91 |
| Flu | y = 73900 (± 3800) – 18200 (± 3100)x_2_ | 0.91 |
| Phe | y = 215000 (± 14000) + 29000 (± 11000)x_3_ | 0.91 |
| Ant | y = 330000 (± 14000) + 66000 (± 11000)x_2_ + 45000 (± 11000)x_3_ – 30000 (± 12000)x_2_x_3_ | 0.75 |
| Flt | y = 350000 (± 11000) + 140000 (± 9700)x_2_ + 90000 (± 9700)x_3_ + 38000 (± 15000)x_3_^2^ | 0.54 |
| Py | y= 380000 (± 9300) + 170000 (± 7700)x_2_ + 100000 (± 7700)x_3_ + 38000 (± 14000)x_2_^2^ + 33000 (± 14000)x_3_^2^ | 0.53 |
| BaA | y = 140000 (± 13000) + 60000 (± 12000)x_2_ + 70000 (± 12000)x_3_ + 71000(± 18000)x_2_^2^ | 0.57 |
| Chr | y = 39000 (± 20000) + 120000 (± 16000)x_2_ + 13000 (± 16000)x_3_ – 72000 (± 29000)x_1_^2^ + 140000 (± 29000)x_2_^2^ | 0.76 |
| BbF | y = 130000 (±23000) + 46000 (± 15000)x_3_ | 0.91 |
| BkF | y = 470000 (± 68000) + 150000 (± 45000)x_3_ | 0.91 |
| BaP | y = 120000 (± 17000) + 44000 (± 14000)x_3_ + 90000 (± 26000)x_2_^2^ | 0.91 |
| InPy | y =130000 (±20000) + 43000 (± 13000)x_3_ | 0.91 |
| DiahA | y = 130000 (± 21000) + 41000 (± 14000)x_3_ – 69000 (± 27000)x_1_^2^ | 0.91 |
| BghiP | y = 170000 (± 18000) + 48000 (± 12000)x_3_ – 69000 (±23000)x_1_^2^ | 0.91 |

x_1_: desorption time

x_2_: extraction temperature

x_3_: extraction time

**Table S6** Comparison of LOD values achieved in this work with previous studies in which CDs or CNTs were used as sorbent material

| **Extraction technique** | **Material** | **Detection technique** | **LOD range** (ng/L) | **Sample volume**  (mL) | **Desorption** | **Re-use**  (times) | **Ref** |
| --- | --- | --- | --- | --- | --- | --- | --- |
| SPME | CDs/MWCNTs | GC-MS | 0.1-0.7 | 19.5 | TD | 50 | this study |
| SPE | CDs/microporous silica | LC-Flu | 200-3000 | 50 | Hex/MeOH (4+4 mL) | - | [14] |
| MDSPE | CDs/GO | GC-FID | 100-500 | 20 | Tol (300 μL) | 10 | [15] |
| SPE | CDs/microporous silica | LC-Flu | 30-800 | 50 | Hex/MeOH (4+4 mL) | - | [16] |
| DSPE | MWCNTs | GC-MS | 3-30 | 250 | Tol (8 mL) | - | [17] |
| SPE | CDs/microporous silica | LC-Flu | 1.2-38 | 10 | MeOH (4mL) | - | [18] |
| DSPE | Sulfur-Coated Magnetic CNTs | LC-UV | 50-110 | 100 | ACN (2mL) | - | [19] |
| MDSPE | MWCNTs/MOF | LC-UV | 20-410 | 15 | ACN (1.5 mL) | 9 | [20] |
| MDSPE | CDs/MWCNTs | GC-FID | 600-3000 | 10 | Tol (200 μL) | 12 | [21] |
| SBSE | PDMS/CDs/DVB | LC-UV | 13-103 | 10 | MeOH (90 μL) | 40 | [22] |
| SPME | MWCNTs | GC-MS | 0.1-1.2 | 15 | TD | - | [23] |
| μSPE | gellan gum/ MWCNTs | LC-UV | 10-60 | 40 | THF (100 μL) | - | [24] |
| SPME | KBF/MWCNTs | GC-MS | 40-120 | 20 | TD | 80 | [25] |
| SPME | Aramid-Wrapped MWCNTs/silica | GC-MS | 100-300 | 10 | TD | 180 | [26] |
| SPME | PANI/MWCNTs /MOF | LC-UV | 10 | 10 | MeOH (300 μL) | - | [27] |
| In-tube SPME | CNTs/mesoporous silica | LC-UV | 5-50 | 70 | Eluting solution | >100 | [28] |
| DSPE | Cyclodextrin/ starch | GC-FID | 10-70 | 10 | Tol (100 μL) | - | [29] |
| SPME | PANI/MWCNTs /MOF | GC-FID | 0.3-0.8 | 10 | TD | - | [30] |
| SPE | SGL/MWCNT/ cellulose triacetate | LC-UV | 20-90 | 7.5 | DCM/acetone (1+1 mL) | 8 | [31] |

ACN: acetonitrile; DCM: dichloromethane; DSPE: dispersive solid phase extraction; GC-FID: gas chromatography-flame ionization detection; GC-MS: gas chromatography-mass spectrometry; GO: graphene oxide; Hex: hexane; KBF: benzene-constructed porous organic polymer; LC-Flu: liquid chromatography-fluorescence detection; LC-UV: liquid chromatography-Ultraviolet detection; MDSPE: magnetic dispersive solid phase extraction; MeOH: methanol; MOF: magnetic organic frameworks; PANI: polyaniline; SBSE: stir bar sorptive extraction; SGL: single layer graphene; SPE: solid phase extraction; μSPE: micro-solid phase extraction; SPME: solid phase microextraction; TD: thermal desorption; THF: tetrahydrofuran; Tol: toluene.

**Table S7** Repeatability and intermediate precision of the MWCNT-H_2_O_2_-γ-CD-SPME-GC-MS method (n=6)

|  | **Repeatability (RSD)** | | | **Intermediate precision (RSD)** | | |
| --- | --- | --- | --- | --- | --- | --- |
| **PAHs** | 2 ng/L | 15 ng/L | 30 ng/L | 2 ng/L | 15 ng/L | 30 ng/L |
| Nap | 11 | 7 | 4 | 20 | 17 | 18 |
| Acy | 7 | 6 | 5 | 16 | 11 | 10 |
| Ace | 14 | 11 | 8 | 13 | 18 | 14 |
| Flu | 14 | 5 | 7 | 13 | 9 | 10 |
| Phe | 17 | 14 | 9 | 20 | 18 | 8 |
| Ant | 17 | 10 | 8 | 19 | 10 | 7 |
| Flt | 13 | 12 | 8 | 16 | 17 | 12 |
| Py | 11 | 11 | 9 | 16 | 18 | 14 |
| BaA | 7 | 11 | 5 | 18 | 13 | 11 |
| Chr | 14 | 2 | 3 | 21 | 13 | 8 |
| BbF | 6 | 13 | 4 | 14 | 20 | 7 |
| BkF | 20 | 11 | 7 | 18 | 13 | 6 |
| BaP | 19 | 11 | 5 | 20 | 13 | 17 |
| InPy | 14 | 11 | 7 | 11 | 17 | 10 |
| DiahA | 18 | 5 | 5 | 18 | 18 | 15 |
| BghiP | 18 | 6 | 3 | 17 | 16 | 13 |

n.q.: not quantitated (<LOQ)

**Table S8** Recovery rates (n=10) of the MWCNT-H_2_O_2_-γ-CD-SPME-GC-MS method

| **PAHs** | **RR** (±st.dev.) | | |
| --- | --- | --- | --- |
|  | **3 ng/L** | **10 ng L^-1^** | **20 ng L^-1^** |
| Nap | 87.8 (±0.2) | 91.0 (±0.1) | 91.45 (±0.26) |
| Acy | 111.31 (±0.04) | 94.3 (±0.1) | 91.19 (±0.18) |
| Ace | 110.57 (±0.06) | 91.1 (±0.1) | 91.87 (±0.29) |
| Flu | 109.9 (±0.1) | 95.3 (±0.2) | 94.85 (±0.29) |
| Phe | 112.72 (±0.07) | 119.7 (±0.1) | 117.56 (±0.19) |
| Ant | 89.1 (±0.1) | 90.4 (±0.1) | 91.7 (±0.1) |
| Flt | 119.80 (±0.04) | 90.80 (±0.04) | 97.8 (±0.3) |
| Py | 90.38 (±0.06) | 92.26 (±0.03) | 97.28 (±0.39) |
| BaA | 96.17 (±0.06) | 95.7 (±0.1) | 98.8 (±0.5) |
| Chr | 119.33 (±0.01) | 93.40 (±0.04) | 96.11 (±0.18) |
| BbF | 104.16 (±0.08) | 105.1 (±0.1) | 105.2 (±0.2) |
| BkF | 107.15 (±0.03) | 97.7 (±0.1) | 100.9 (±0.3) |
| BaP | 95.51 (±0.03) | 91.4 (±0.1) | 94.1 (±0.2) |
| InPy | 109.07 (±0.18) | 100.56 (±0.19) | 97.98 (±0.16) |
| DiahA | 115.19 (±0.09) | 90.11 (±0.9) | 99.01 (±0.02) |
| BghiP | 90.7 (±0.3) | 88.09 (±0.05) | 90.3 (±0.1) |


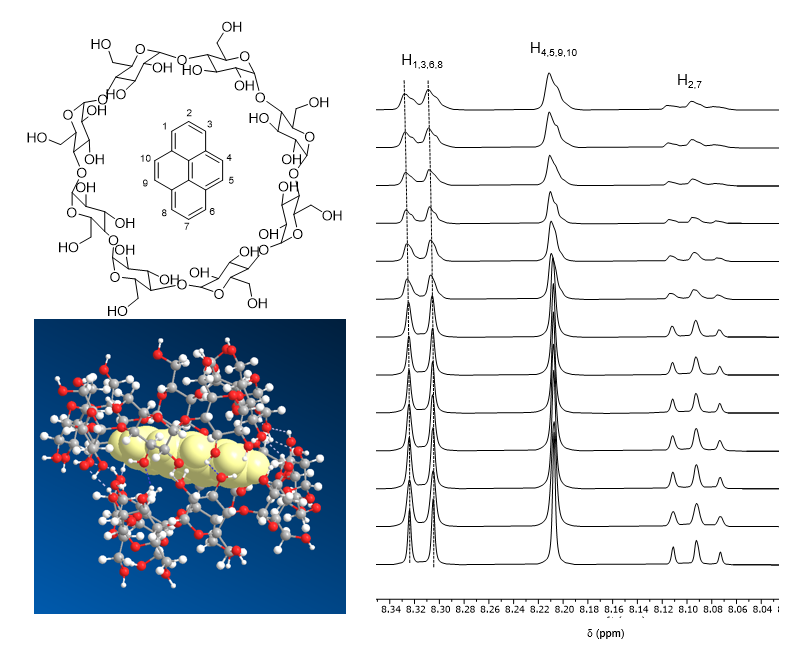


**Fig. S3.** ^1^H-NMR stack plot (expanded aromatic region, 400 MHz) of the titration experiment of a solution of Py in DMSO-d_6_ (c = 10^-3^ M) with an increasing amount of a solution of γ-CD (c = 10^-3^ M) in the same solvent. At the bottom is the spectrum of the free Py, and at the top is the spectrum for a 1:0.25 Py/ γ-CD solution. Pyrene protons labelling is indicated in the sketch on the left, while the PM3 minimised structure of the 2:1 host/guest adduct is represented beneath.

**Fig. S4**. Isotherm titration fluorescence emission curve (λ 372 nm) of pyrene with γ-CD


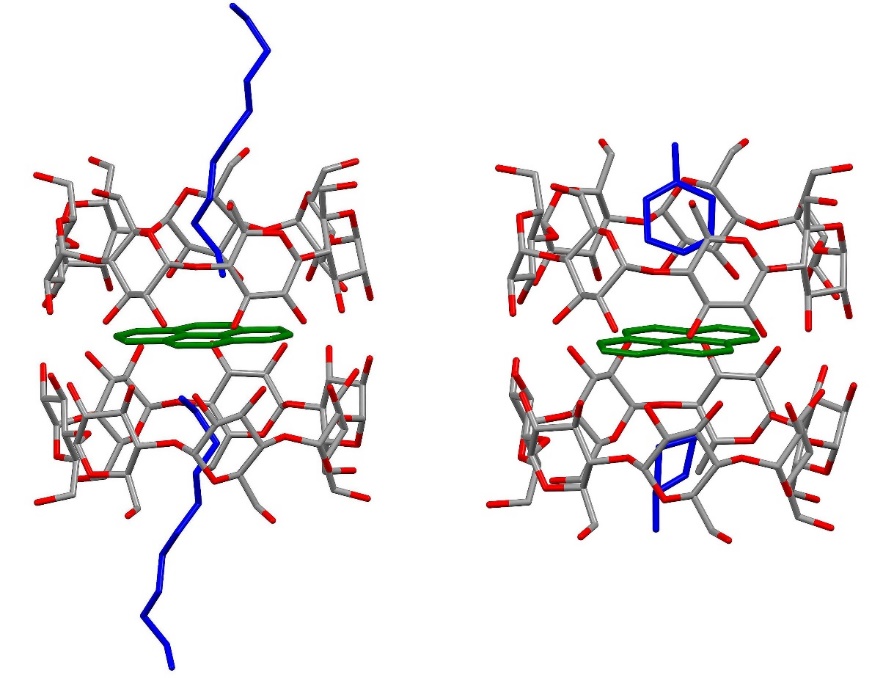


**Fig. S5** Perspective view of the crystal structure of PUKPIU (left) and PUKPOA (right). Alcohol molecules and pyrene units are represented in blue and green, respectively. Hydrogen atoms and lattice solvent molecules have been omitted for clarity.


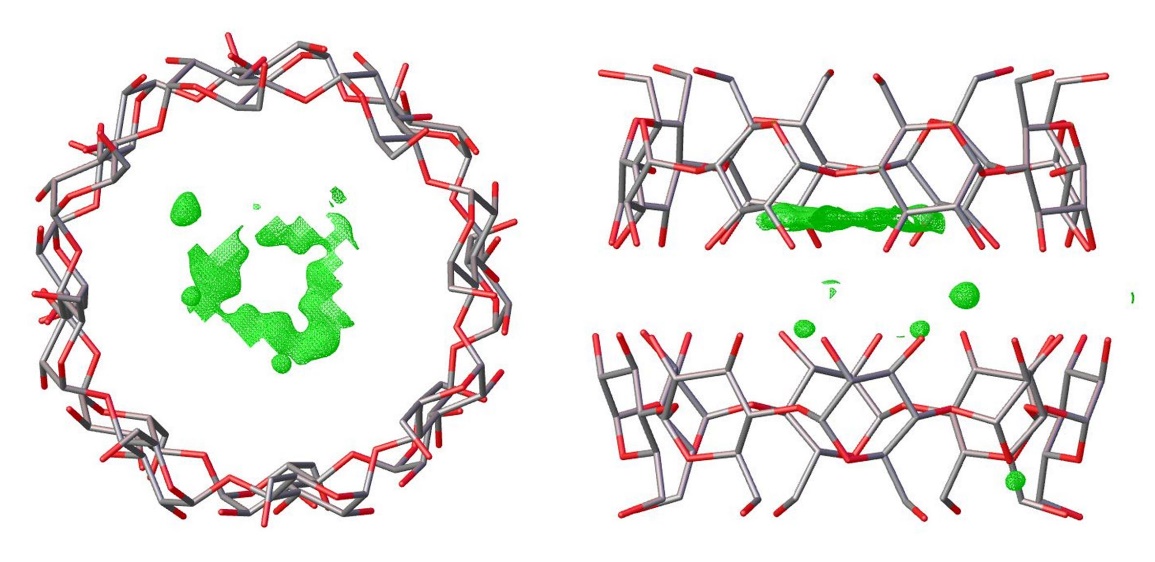


**Fig. S6**. Top (left) and side (right) view of the crystal structure obtained by mixing solutions of γ-cyclodextrin and pyrene. Hydrogen atoms and lattice water molecules are not shown for clarity. The residual electron density is represented as a green mesh.

**References**

1. Carlson, R. *Design and Optimization in Organic Synthesis*; Elsevier: Amsterdam, 1992; ISBN 9780080868356.

2. Bianchi, F.; Pankajakshan, A.; Fornari, F.; Mandal, S.; Pelagatti, P.; Bacchi, A.; Mazzeo, P.P.; Careri, M. A Zinc Mixed-Ligand Microporous Metal-Organic Framework as Solid-Phase Microextraction Coating for Priority Polycyclic Aromatic Hydrocarbons from Water Samples. *Microchem. J.* **2020**, *154*, 104646, doi:10.1016/j.microc.2020.104646.

3. Bianchi, F.; Agazzi, S.; Riboni, N.; Erdal, N.; Hakkarainen, M.; Ilag, L.L.; Anzillotti, L.; Andreoli, R.; Marezza, F.; Moroni, F.; et al. Novel Sample-Substrates for the Determination of New Psychoactive Substances in Oral Fluid by Desorption Electrospray Ionization-High Resolution Mass Spectrometry. *Talanta* **2019**, *202*, 136–144, doi:10.1016/j.talanta.2019.04.057.

4. Riboni, N.; Fornari, F.; Bianchi, F.; Careri, M. A Simple and Efficient Solid-Phase Microextraction – Gas Chromatography – Mass Spectrometry Method for the Determination of Fragrance Materials at Ultra-Trace Levels in Water Samples Using Multi-Walled Carbon Nanotubes as Innovative Coating. *Talanta* **2021**, *224*, 121891, doi:10.1016/j.talanta.2020.121891.

5. Riboni, N.; Trzcinski, J.W.; Bianchi, F.; Massera, C.; Pinalli, R.; Sidisky, L.; Dalcanale, E.; Careri, M. Conformationally Blocked Quinoxaline Cavitand as Solid-Phase Microextraction Coating for the Selective Detection of BTEX in Air. *Anal. Chim. Acta* **2016**, *905*, 79–84, doi:10.1016/j.aca.2015.12.005.

6. Levine, M.; Smith, B.R. Enhanced Characterization of Pyrene Binding in Mixed Cyclodextrin Systems via Fluorescence Spectroscopy. *J. Fluoresc.* **2020**, *30*, 1015–1023, doi:10.1007/s10895-020-02572-5.

7. Dyck, A.S.M.; Kisiel, U.; Bohne, C. Dynamics for the Assembly of Pyrene−γ-Cyclodextrin Host−Guest Complexes. *J. Phys. Chem. B* **2003**, *107*, 11652–11659, doi:10.1021/jp035544c.

8. AXS, S.B.; Madison, W. USA, 2004; SAINT, Software Users Guide, Version 6.0; Bruker Analytical X-Ray Systems. *Softw. Users Guid. Version 6.0; Bruker Anal. X-ray Syst.* **1999**.

9. Sheldrick, G.M. SADABS: Area-Detector Absorption Correction, v2. 10. *Univ. Göttingen, Ger.* **1999**.

10. Burla, M.C.; Caliandro, R.; Carrozzini, B.; Cascarano, G.L.; Cuocci, C.; Giacovazzo, C.; Mallamo, M.; Mazzone, A.; Polidori, G. Crystal Structure Determination and Refinement via SIR2014. *J. Appl. Crystallogr.* **2015**, *48*, 306–309, doi:10.1107/S1600576715001132.

11. Sheldrick, G.M. A Short History of SHELX. *Acta Crystallogr. Sect. A Found. Crystallogr.* **2008**, *64*, 112–122, doi:10.1107/S0108767307043930.

12. Sheldrick, G.M. Crystal Structure Refinement with SHELXL. *Acta Crystallogr. Sect. C Struct. Chem.* **2015**, *71*, 3–8, doi:10.1107/S2053229614024218.

13. Farrugia, L.J. WinGX Suite for Small-Molecule Single-Crystal Crystallography. *J. Appl. Crystallogr.* **1999**, *32*, 837–838, doi:10.1107/S0021889899006020.

14. Belenguer-Sapiña, C.; Pellicer-Castell, E.; El Haskouri, J.; Guillem, C.; Simó-Alfonso, E.F.; Amorós, P.; Mauri-Aucejo, A. Design, Characterization and Comparison of Materials Based on β and γ Cyclodextrin Covalently Connected to Microporous Silica for Environmental Analysis. *J. Chromatogr. A* **2018**, *1563*, 10–19, doi:10.1016/j.chroma.2018.05.070.

15. Majd, M.; Nojavan, S. Determination of Polycyclic Aromatic Hydrocarbons in Soil, Tree Leaves, and Water Samples by Magnetic Dispersive Solid-Phase Extraction Based on β-Cyclodextrin Functionalized Graphene Oxide Followed by GC-FID. *Microchem. J.* **2021**, *171*, 106852, doi:10.1016/j.microc.2021.106852.

16. Mauri-Aucejo, A.; Amorós, P.; Moragues, A.; Guillem, C.; Belenguer-Sapiña, C. Comparison of the Solid-Phase Extraction Efficiency of a Bounded and an Included Cyclodextrin-Silica Microporous Composite for Polycyclic Aromatic Hydrocarbons Determination in Water Samples. *Talanta* **2016**, *156*–*157*, 95–103, doi:10.1016/j.talanta.2016.05.011.

17. Paszkiewicz, M.; Caban, M.; Bielicka-Giełdoń, A.; Stepnowski, P. Optimization of a Procedure for the Simultaneous Extraction of Polycyclic Aromatic Hydrocarbons and Metal Ions by Functionalized and Non-Functionalized Carbon Nanotubes as Effective Sorbents. *Talanta* **2017**, *165*, 405–411, doi:10.1016/j.talanta.2016.10.049.

18. Soler-Seguí, S.; Belenguer-Sapiña, C.; Amorós, P.; Mauri-Aucejo, A. Evaluation of a Cyclodextrin-Silica Hybrid Microporous Composite for the Solid-Phase Extraction of Polycyclic Aromatic Hydrocarbons. *Anal. Sci.* **2016**, *32*, 659–665, doi:10.2116/analsci.32.659.

19. Sun, W.; Hong, Y.; Li, T.; Chu, H.; Liu, J.; Feng, L. Application of Sulfur-Coated Magnetic Carbon Nanotubes for Extraction of Some Polycyclic Aromatic Hydrocarbons from Water Resources. *Chemosphere* **2022**, *309*, 136632, doi:10.1016/j.chemosphere.2022.136632.

20. Yang, J.; Zhang, X.; Wang, X.; Wang, H.; Zhao, J.; Zhou, Z.; Du, X.; Lu, X. In Situ Anchor of Multi-Walled Carbon Nanotubes into Iron-Based Metal-Organic Frameworks for Enhanced Adsorption of Polycyclic Aromatic Hydrocarbons by Magnetic Solid-Phase Extraction. *J. Chromatogr. A* **2022**, *1681*, 463459, doi:10.1016/j.chroma.2022.463459.

21. Yazdanpanah, M.; Nojavan, S. Micro-Solid Phase Extraction of Some Polycyclic Aromatic Hydrocarbons from Environmental Water Samples Using Magnetic β-Cyclodextrin-Carbon Nano-Tube Composite as a Sorbent. *J. Chromatogr. A* **2019**, *1585*, 34–45, doi:10.1016/j.chroma.2018.11.066.

22. Yu, C.; Yao, Z.; Hu, B. Preparation of Polydimethylsiloxane/β-Cyclodextrin/Divinylbenzene Coated “Dumbbell-Shaped” Stir Bar and Its Application to the Analysis of Polycyclic Aromatic Hydrocarbons and Polycyclic Aromatic Sulfur Heterocycles Compounds in Lake Water and Soil by Hig. *Anal. Chim. Acta* **2009**, *641*, 75–82, doi:10.1016/j.aca.2009.03.031.

23. Arcoleo, A.; Bianchi, F.; Careri, M. Helical Multi-Walled Carbon Nanotube-Coated Fibers for Solid-Phase Microextraction Determination of Polycyclic Aromatic Hydrocarbons at Ultra-Trace Levels in Ice and Snow Samples. *J. Chromatogr. A* **2020**, *1631*, 461589, doi:10.1016/j.chroma.2020.461589.

24. Zulkipli, N.A.; Khalik, W.M.A.W.M.; Ariffin, M.M.; Aboul-Enein, H.Y.; Yahaya, N.; Kamaruzaman, S.; Loh, S.H. Multiwalled Carbon Nanotubes-Encapsulated Gellan Gum Membrane for Micro-Solid Phase Extraction of Selected Polycyclic Aromatic Hydrocarbons in Environmental Water and Beverages. *Chromatographia* **2022**, *85*, 23–33, doi:10.1007/s10337-021-04102-2.

25. Li, J.; Xiao, Z.; Wang, W.; Zhang, S.; Wu, Q.; Wang, C.; Wang, Z. Rational Integration of Porous Organic Polymer and Multiwall Carbon Nanotube for the Microextraction of Polycyclic Aromatic Hydrocarbons. *Microchim. Acta* **2020**, *187*, doi:10.1007/s00604-020-04261-3.

26. Alhendal, A.; Almoaeen, R.A.; Rashad, M.; Husain, A.; Mouffouk, F.; Ahmad, Z. Aramid-Wrapped CNT Hybrid Sol–Gel Sorbent for Polycyclic Aromatic Hydrocarbons. *RSC Adv.* **2022**, *12*, 18077–18083, doi:10.1039/D2RA02659G.

27. Chen, J.; Zhang, B.; Dang, X.; Zheng, D.; Ai, Y.; Chen, H. A Nanocomposite Consisting of Etched Multiwalled Carbon Nanotubes, Amino-Modified Metal-Organic Framework UiO-66 and Polyaniline for Preconcentration of Polycyclic Aromatic Hydrocarbons Prior to Their Determination by HPLC. *Microchim. Acta* **2020**, *187*, 78, doi:10.1007/s00604-019-3997-1.

28. Loussala, H.M.; Feng, J.; Han, S.; Sun, M.; Ji, X.; Li, C.; Fan, J.; Pei, M. Carbon Nanotubes Functionalized Mesoporous Silica for In‐tube Solid‐phase Microextraction of Polycyclic Aromatic Hydrocarbons. *J. Sep. Sci.* **2020**, *43*, 3275–3284, doi:10.1002/jssc.202000047.

29. Yazdanpanah, M.; Nojavan, S. Cyclodextrin-Starch Hard Gel as an Efficient Green Sorbent for Dispersive Micro Solid-Phase Extraction of Eight Polycyclic Aromatic Hydrocarbons from Environmental Water Samples. *Microchem. J.* **2021**, *168*, 106509, doi:10.1016/j.microc.2021.106509.

30. Hajializadeh, A.; Ansari, M.; Foroughi, M.M.; Kazemipour, M. Ultrasonic Assisted Synthesis of a Novel Ternary Nanocomposite Based on Carbon Nanotubes/Zeolitic Imidazolate Framework-67/Polyaniline for Solid-Phase Microextraction of Organic Pollutants. *Microchem. J.* **2020**, *157*, 105008, doi:10.1016/j.microc.2020.105008.

31. Mukhtar, N.H.; See, H.H. Carbonaceous Nanomaterials Immobilised Mixed Matrix Membrane Microextraction for the Determination of Polycyclic Aromatic Hydrocarbons in Sewage Pond Water Samples. *Anal. Chim. Acta* **2016**, *931*, 57–63, doi:10.1016/j.aca.2016.04.032.
